# Discovery and confirmation of crucial genes associated with radiation-induced heart disease

**DOI:** 10.7150/ijms.107667

**Published:** 2025-02-18

**Authors:** Chuanbin Liu, Jingqi Shi, Lei Xing, Binwei Yao, Jing Liu, Yiru Wang, Jiao Fan

**Affiliations:** 1 Department of Emergency, the First Medical Center of PLA General Hospital, Beijing, China.; 2 Western Medical Branch of PLA General Hospital, Beijing, China.; 3 Institute of Geriatrics, the Second Medical Centre & National Clinical Research Centre for Geriatric Disease, Chinese PLA General Hospital, Beijing, China.; 4 Institute of Radiation Medicine, Academy of Military Medical Sciences, Beijing, China.; 5 Department of Ultrasound, the First Medical Center, Chinese PLA General Hospital, Beijing, China.

**Keywords:** malignant tumor, radiotherapy, radiation-induced heart disease, systemic inflammation, mitochondrial dysfunction

## Abstract

**Objective:** Radiotherapy is an essential method for treating cancerous tumors, and the resultant radiation-induced heart disease (RIHD) has emerged as the leading non-cancerous cause of mortality among cancer survivors. However, the mechanisms of RIHD are still unknown, and specific biomarkers and effective treatment methods are needing to be found.

**Methods:** Fourteen male C57BL/6J mice, each 8 weeks old, were randomly assigned into two groups: an experimental group (n = 7) and a control group (n = 7). The test group underwent irradiation with 30 Gy of ^60^Co γ-rays. To assess the acute and chronic damage to the myocardium caused by radiation, heart tissues were collected at one day and six weeks after irradiation for transcriptome sequencing, and H&E staining and immunohistochemical staining were done, respectively.

**Results:** One day after radiation, the myocardial tissue showed a significant amount of inflammatory cell infiltration. Following a period of six weeks, there was an increase in hypertrophic cardiomyocytes and myocardial fibrosis. Additionally, we identified several genes (*Cmpk2, Ifit3, Dhx58, Slc2a1, and Thbs1*) that were strongly associated with RIHD. The expression of these genes in heart tissue was significantly upregulated after six weeks of radiation. Findings from the GO functional and KEGG pathway enrichment analysis, along with the hub gene function analysis, indicate that the mechanism behind RIHD might be linked to systemic inflammation and mitochondrial dysfunction.

**Conclusion:** Acute radiation myocardial injury is characterized by inflammation, while chronic radiation myocardial injury is characterized by myocardial fibrosis. RIHD is linked to *Cmpk2*, *Ifit3*, *Dhx58*,* Slc2a1*, and *Thbs1* genes through a mechanism that may cause systemic inflammation and mitochondrial dysfunction.

## Introduction

Studies show that in 2020, there were 19.29 million new cancer diagnoses globally, and cancer-related deaths continue to be a major cause of mortality; breast cancer, lung cancer, and other thoracic tumors represent a significant portion of these cases 1. Despite the swift progress in molecular targeted treatments, radiation therapy continues to play a crucial role in combating tumors and is frequently combined with surgical procedures or chemotherapy 2. However, the radiation produced during radiation therapy can also cause some damage to the heart, resulting in radiation-induced heart disease (RIHD), which includes acute and chronic pericardial disease, cardiomyopathy, valvular insufficiency, arrhythmia, and coronary artery disease (CAD), etc 3, 4. Advances in medical technology have significantly improved the effectiveness of tumor treatment, leading to long-term survival for many patients. RIHD has emerged as the leading non-cancerous cause of mortality among individuals who have survived tumors. This greatly diminishes the survival benefit of radiotherapy and even exceeds the lethality due to recurrence of the tumor itself 5. However, the mechanism of RIHD is still unclear and specific biomarkers and effective treatments are lacking. Therefore, it is important to elucidate the potential mechanisms of RIHD for its early diagnosis and treatment.

Studies have shown that individuals treated with chest radiotherapy face a 2% increased risk of cardiovascular disease within 5 years and a 23% higher risk over 20 years, compared to those who did not undergo such treatment 6. The estimated occurrence of RIHD ranges from 10% to 30% within 5 to 10 years following radiation therapy. This is especially relevant for chest tumors near the heart and major blood vessels, including breast cancer, lung cancer, Hodgkin's lymphoma, and mediastinal cancers, which are more likely to directly inflict cardiac and vascular harm, resulting in RIHD 7, 8. RIHD is strongly associated with radiation dose. In multivariate analyses, higher doses of cardiac radiation were found to increase the risk of death 9. The occurrence of CAD in individuals undergoing radiotherapy can reach up to 85%, strongly linked to the radiation dosage, location, and duration. Patients in this research had an average heart dose of 4.9 Gy, with a 7.4% rise in major coronary artery events for each additional 1 Gy of radiation 10. Furthermore, radiotherapy also increases the risk of arrhythmias such as atrial fibrillation 11. However, the exact process of RIHD remains unclear, it is thought to include harm to vascular endothelial cells, an inflammatory reaction, oxidative stress, mitochondrial and endoplasmic reticulum dysfunction, and the interplay of various cytokines 12-14.

Radiation causes cardiac injury and induces changes in gene transcriptomics, but it remains unclear how acute and chronic injury are similar or different, and which gene expression differences in cardiac tissue are induced by radiation. To clarify the aforementioned questions and elucidate the potential mechanisms of RIHD, we established an animal model of RIHD by irradiating mice with ^60^Co γ-rays, and then obtained the cardiac tissue for RNA-seq analysis on one day and six weeks after irradiation, performed pathological testing. The goal is to investigate the possible mechanisms and potential targets of RIHD, and to provide a reference for early diagnosis and specific intervention of clinical RIHD.

## Methods

### Mice and irradiation

The mice were sourced from Beijing Vital River Laboratory Animal Technology Co., Ltd (Beijing, China). The animal experiment involved fourteen 8-week-old male C57BL/6J mice. All procedures were sanctioned by the Institutional Animal Care and Use Committee (IACUC-AMMS-2020-780), adhering to the guidelines outlined in the Guide for the Care and Use of Laboratory Animals by the US National Institute of Health. The mice were housed individually in cages that were changed daily and were provided with sterilized food and water at a temperature of 25±1°C. In this research, animals were exposed to ^60^Co γ-rays (Institute of Military Medical Sciences, Academy of Military Sciences, Beijing, China). The mice were anesthetized with pentobarbital sodium (40 mg/kg body weight) and then locally irradiated in the anterior chest under general anesthesia. Other animal parts outside chest were covered with 5 mm thick lead block. The study used a radiation dose of 30 Gy with a dose rate of 0.97 Gy/min 15. Fourteen mice were split into two groups: an experimental group (n = 7) that underwent irradiation and a control group (n = 7). Each group was then sacrificed at one day (n = 3) and six weeks (n = 4) post-irradiation, respectively, to collect heart tissue for sequencing and pathological analysis. All seven mice in the experimental group survived the irradiation, resulting in a 100% survival rate. Fourteen cardiac samples from the mice were suitable for sequencing.

**Figure 1:**
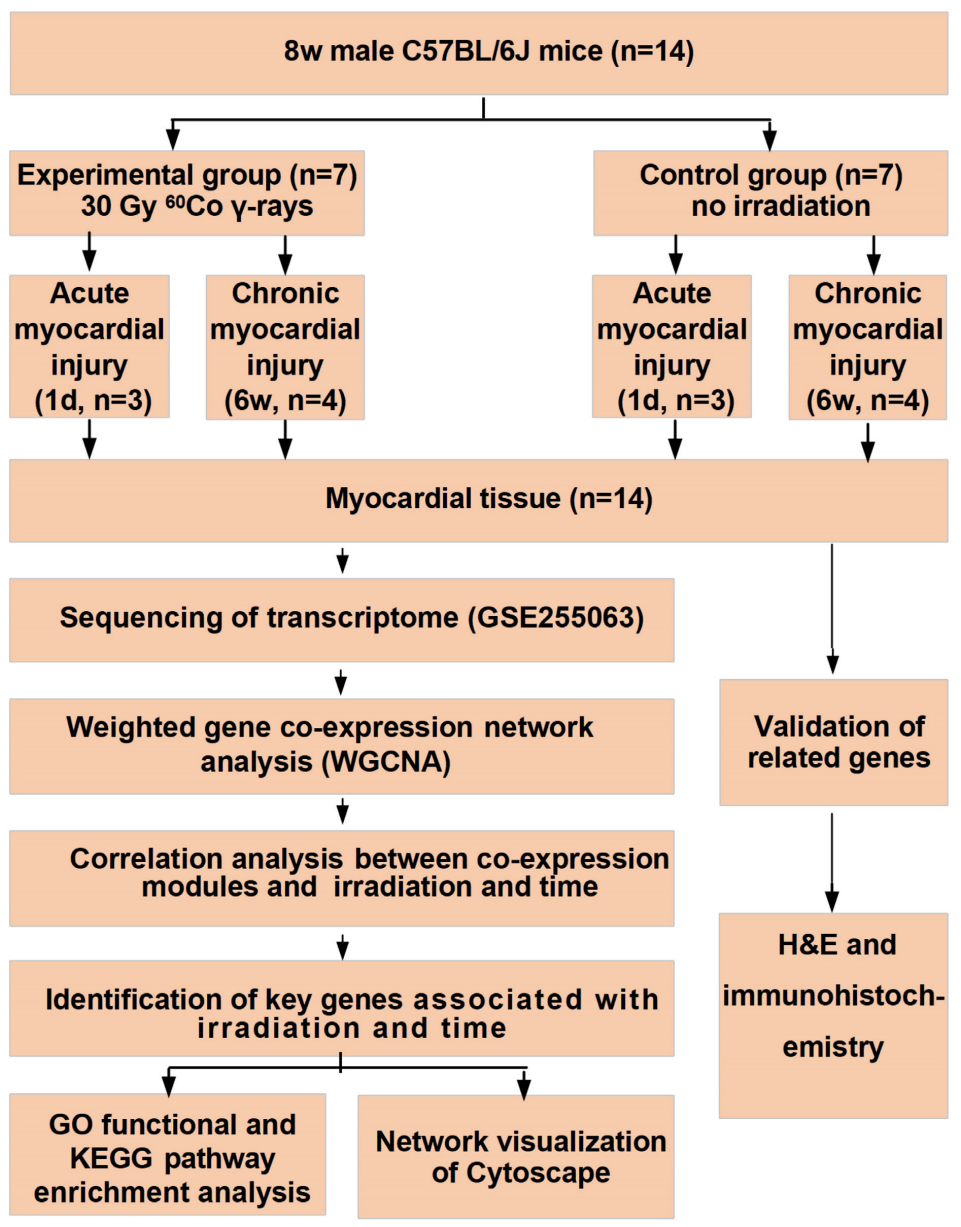
The flowchart of analysis process.

Fig. 1 shows a flow chart of the study.

### Data collection and processing for RNA-sequencing

The standard Illumina protocol was used to prepare the RNA-seq library for sequencing. RNA was extracted from the heart tissues of 14 mice (3 non-irradiated for 1 day, 3 irradiated for 1 day, 4 non-irradiated for 6 weeks, 4 irradiated for 6 weeks) using TRIzol reagent (Invitrogen) and subsequently treated with RNase-free DNase I to eliminate any residual genomic DNA. The samples were then stratified by irradiation and time. Library construction and sequencing data were generated by Novogene (Beijing, China) using a 150-bp paired end Illumina HiSeq platform with RNA quality assessed using an Agilent 2100 Bioanalyzer. RNA-seq data quality control was performed using FastQC (v0.11.8). Trim Galore (v0.6.1) was employed with standard settings for paired-end RNA-seq data to eliminate adapters and low-quality sequences. The RNA-seq data was trimmed and aligned to the mouse mm10 transcriptome using STAR (v2.5.0a) with default parameters. In HTSeq (v0.10.0), gene expression levels were measured in terms of fragments per kilobase of transcript per million mapped reads (FPKM) 16. To identify differentially expressed genes (DEGs), DESeq2 (version 1.40.1) was employed, using criteria of expression fold change greater than 2 or less than 0.5, and a *P*-value below 0.05. The differential expression's statistical significance was evaluated using the Wald test. The outcomes were screened using a false discovery rate threshold of less than 0.05.

### Construction of the weighted gene co-expression network

To build a gene co-expression network, we utilized the WGCNA R package (version 1.72-1) and employed the hclust function to cluster the samples, aiming to identify any outliers. The pickSoftThreshold function was used to evaluate the soft thresholding power β, which varied between 1 and 20, through network topology analysis. We selected power level 11, the smallest β where the scale-free topology fit index achieved an R^2^ threshold of 0.9.

### Selection of key modules corresponding to radiation

Gene modules consist of groups of genes that are expressed at similar levels. WGCNA employs a hierarchical clustering method to detect gene modules, which are subsequently depicted in various colors. Any genes that are not assigned to a module are placed in the grey module. For each module, principal component analysis (PCA) was conducted, and the module eigengenes (MEs) were derived from the first principal component, reflecting the module's aggregate expression level 17. The study calculated the Pearson correlation between MEs and radiation, as well as dose and student asymptotic *P*-value, to create a heat map showing the association between modules and traits. Gene clusters closely linked to the traits of interest were identified based on correlation and module significance (*P*<0.05) 17. In the intramodular analysis, the research determined the gene significance (GS) and module membership (MM) values. The GS metric indicates the relationship between gene expression and traits in the computed module, whereas the MM metric signifies the correlation between a gene's expression and the principal component's expression within that module 17. The essential core genes for the target modules were identified by defining the value ranges for GS, MM, and the *q*-value of trait association, calculated via the NetworkScreening function.

### Network visualization and functional enrichment study

The core gene networks of the modules of interest were exported to Cytoscape (v3.10.0). To identify the biological roles and potential pathways of genes in trait-associated modules, gene functional annotation was conducted in the primary module using the Gene Ontology (GO) database (http://www.geneontology.org/) and the Kyoto Encyclopedia of Genes and Genomes (KEGG) database (http://www.genome.jp/kegg/).

### Hematoxylin and Eosin (H&E) staining

Following localized radiation treatment, the mice were euthanized, and their cardiac tissues were extracted and preserved. The heart tissues were then fixed in a 10% neutral buffered formalin solution for at least one week and embedded in paraffin. Sections of the heart tissue, 3 µm in size, were stained with H&E (Sinopharm Chemical Reagent Beijing Co., Ltd.). Following dehydration using an alcohol gradient, xylene clearance and cover slips were performed. Observations were performed using a LEICA DM6000 light microscope (Leica, Germany).

### Immunohistochemistry

For antigen retrieval in immunohistochemistry, the slides were heated in a citrate solution with a pH of 6.0. Then, endogenous peroxidase was blocked using hydrogen peroxide/PBS. The primary antibody was incubated at 4°C overnight. The secondary antibody was incubated at room temperature for 2 hours. Blocking was achieved using immunoglobulin from the same species as the secondary antibody (Servicebio, China, 1:200 dilution). The main antibodies employed included rabbit anti-Cmpk2 (Proteintech, USA, 1:200 dilution), rabbit anti-Ifit3 (Proteintech, USA, 1:200 dilution), rabbit anti-Dhx58 (Proteintech, USA, 1:50 dilution), rabbit anti-Slc2a1 (Proteintech, USA, 1:1000 dilution), and rabbit anti-Thbs1 (Proteintech, USA, 1:200 dilution). Negative controls were incorporated, involving the exclusion of the primary antibody and the application of either nonimmune IgG or just the secondary antibody. All negative controls showed insignificant staining. The relevant secondary antibodies conjugated to peroxidase-labeled dextran polymer were used to probe the sections. The sections were then visualized using the diaminobezidin 3 (DAB) system. Pictures were taken with a LEICA DM6000 light microscope (Leica, Germany), and analysis was conducted using Image-Pro Plus Software. We utilized images acquired at a magnification of 40x, and for each mouse, we quantified at least six distinct fields of view.

### Statistical analysis

Data analysis was performed with SPSS software (version21.0; SPSS Inc., Chicago, USA) and R programming language (v4.3.0). Continuous variables were shown as mean ± standard error (SE), while categorical data were represented as percentages. Statistical significance was considered at *P* < 0.05.

## Results

### Differential gene expression with irradiation and time

**Figure 2:**
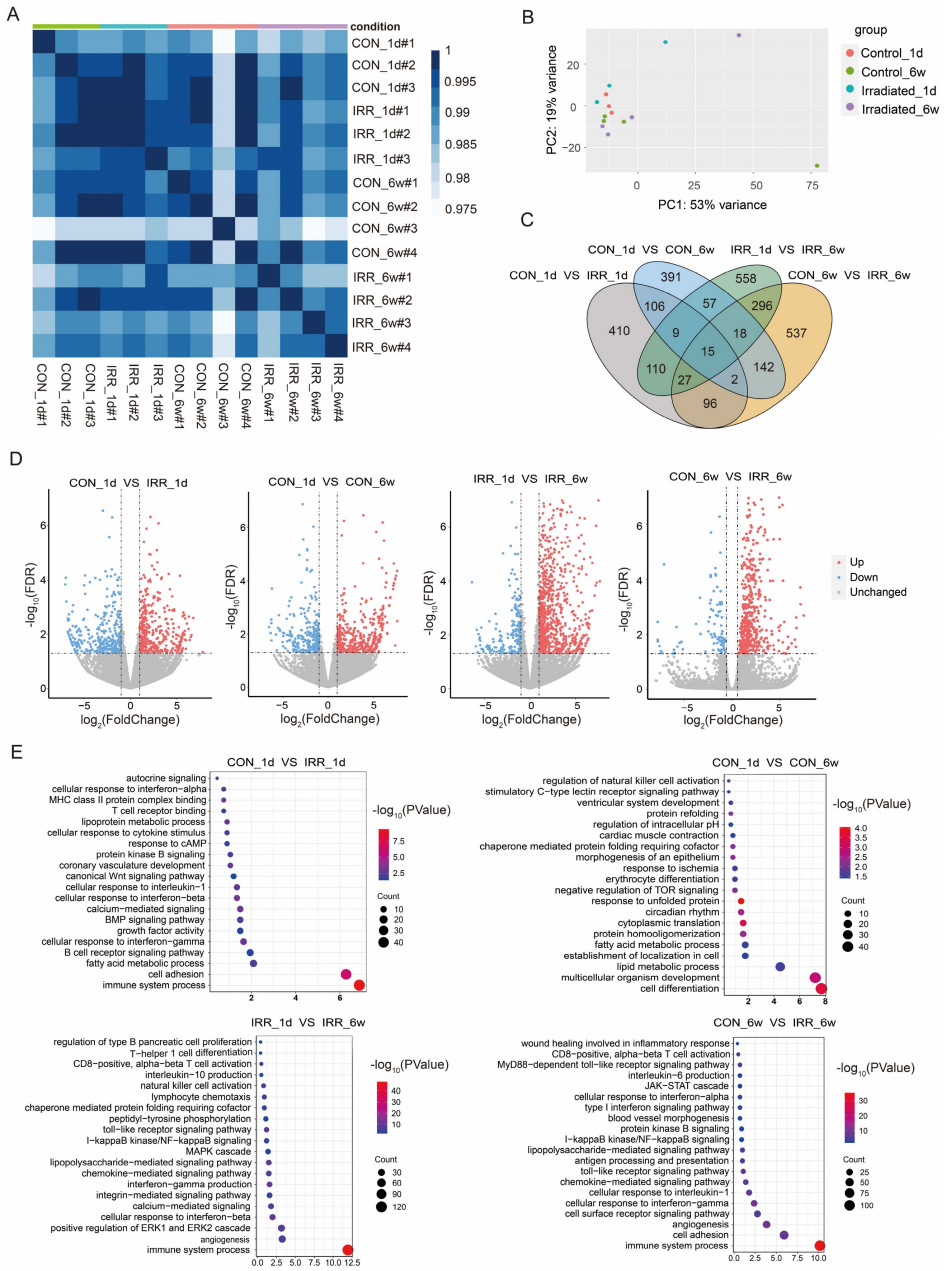
Differential gene expression and pathway enrichment analysis. **A** A heatmap of the pairwise Pearson's correlation between each sample. **B** PCA plot that separates samples from each group. **C** Wayne diagram of differential expression in the irradiated group *vs.* control groups at one day and six weeks after irradiation. **D** Volcano diagram of DEGs in the irradiated group *vs.* control groups at one day and six weeks after irradiation. Red dots and blue dots represent up-regulated and down-regulated immune genes in the irradiated group, respectively. **E** GO function enrichment analysis of DEGs between control and experimental groups at one day and six weeks after irradiation, where the size of the dots represents the number of genes, and the color of the dots represents the -log_10_(*P*-value).

We established an experimental model of cardiac irradiation in rats and subsequently performed transcriptome sequencing analysis. Sample clustering and phenotypic heat map analysis were performed on the gene expression data from all samples 17. Fig. 2A indicates the absence of any notable outlier samples. Therefore, every sample was incorporated into the following data analysis. Correlation analysis of samples revealed good intra-group agreement and large inter-group differences (Fig. 2B). Subsequently, differential gene expression analysis was performed to compare (Fig. 2C and D). Control_1d *vs*. IRR_1d: 778 DEGs, 436 up regulated, 342 down regulated; Control_6w *vs*. IRR_6w: 1,133 DEGs, 822 up regulated, 311 down regulated; Control_1d *vs*. Control_6w: 740 DEGs, 449 up regulated, 291 down regulated; IRR_1d *vs*. IRR_6w: 1,090 DEGs, 883 up regulated, 207 down regulated (Supplementary Table S1). The DEGs were then subjected to GO enrichment analysis, as shown in Fig. 2E and Supplementary Table S2. The gene activities between Control_1d and IRR_1d were significantly associated with immune system activities (GO 0002376), cellular adhesion (GO 0007155), and fatty acid metabolism (GO 0006631). The gene activities between Control_1d and Control_6w were significantly associated with cell differentiation (GO 0030154), development of multicellular organisms (GO 0007275), and lipid metabolism (GO 0006629). The gene activities in IRR_1d compared to IRR_6w were significantly involved in immune responses (GO 0002376), blood vessel formation (GO 0001525), and the enhancement of the ERK1/ERK2 signaling pathway (GO 0070374). Likewise, the gene roles in Control_6w compared to IRR_6w were predominantly associated with immune responses (GO 0002376), cellular adhesion (GO 0007155), and the formation of new blood vessels (GO 0001525). The KEGG pathway analysis indicated that the comparison between Control_1d and IRR_1d showed significant enrichment in hematopoietic cell lineage (mmu04640), p53 signaling pathway (mmu04115), and cell adhesion molecules (mmu04514). The Control_6w compared to IRR_6w showed enrichment in cytokine-cytokine receptor interaction (mmu04060), p53 signaling pathway (mmu04115), and TNF signaling pathway (mmu04668) (Supplementary Table S3).

**Figure 3:**
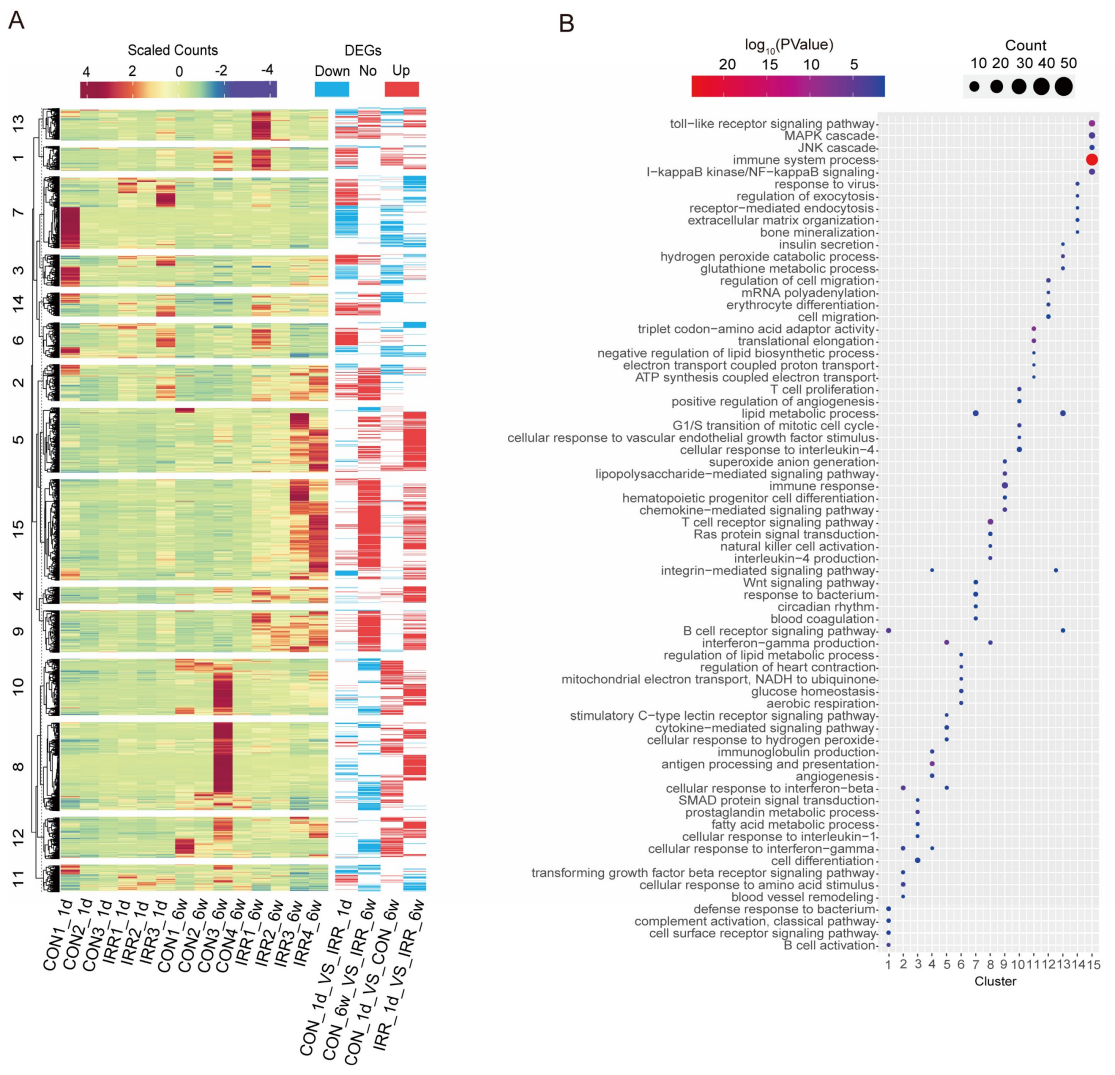
Heat map and DEGs obtained by pairwise comparison. **A** Clustering analysis of DEGs, and k-means clusters (k=15) are marked on the left. **B** The log_10_(*P*-value) of 15 clusters.

The 2,774 DEGs identified in the four groups were combined and subjected to k-means clustering analysis, resulting in 15 categories. Genes in cluster 9 and cluster 15 exhibited high expression levels in the irradiated group (Fig. 3A), indicating a strong association with radiation. Conversely, genes in cluster 5 and cluster 8 showed high expression levels 6 weeks after irradiation, suggesting a correlation with long-term radiation exposure. Fig. 3B shows that cluster 15 is associated with immune system processes and toll-like receptor signaling pathways, including immune-related genes such as *Ccl12* and *Ifi204*. Meanwhile, cluster 9 (including *Igsf6* and *Smpd3*) is associated with immune response and chemokine-mediated signaling pathways. Cluster 8 includes genes related to immunity, like *Kif4* and *Arhgap45*, linked to the T cell receptor signaling pathway and the production of interleukin-4. Meanwhile, cluster 5 is associated with cytokine-mediated signaling pathways, including some apoptosis-related genes such as *Aif1* and *Trpm2*. These findings suggest that inflammation and apoptosis play a role in acute and chronic radiation myocardial injury.

### Construction of WGCNA network

**Figure 4:**
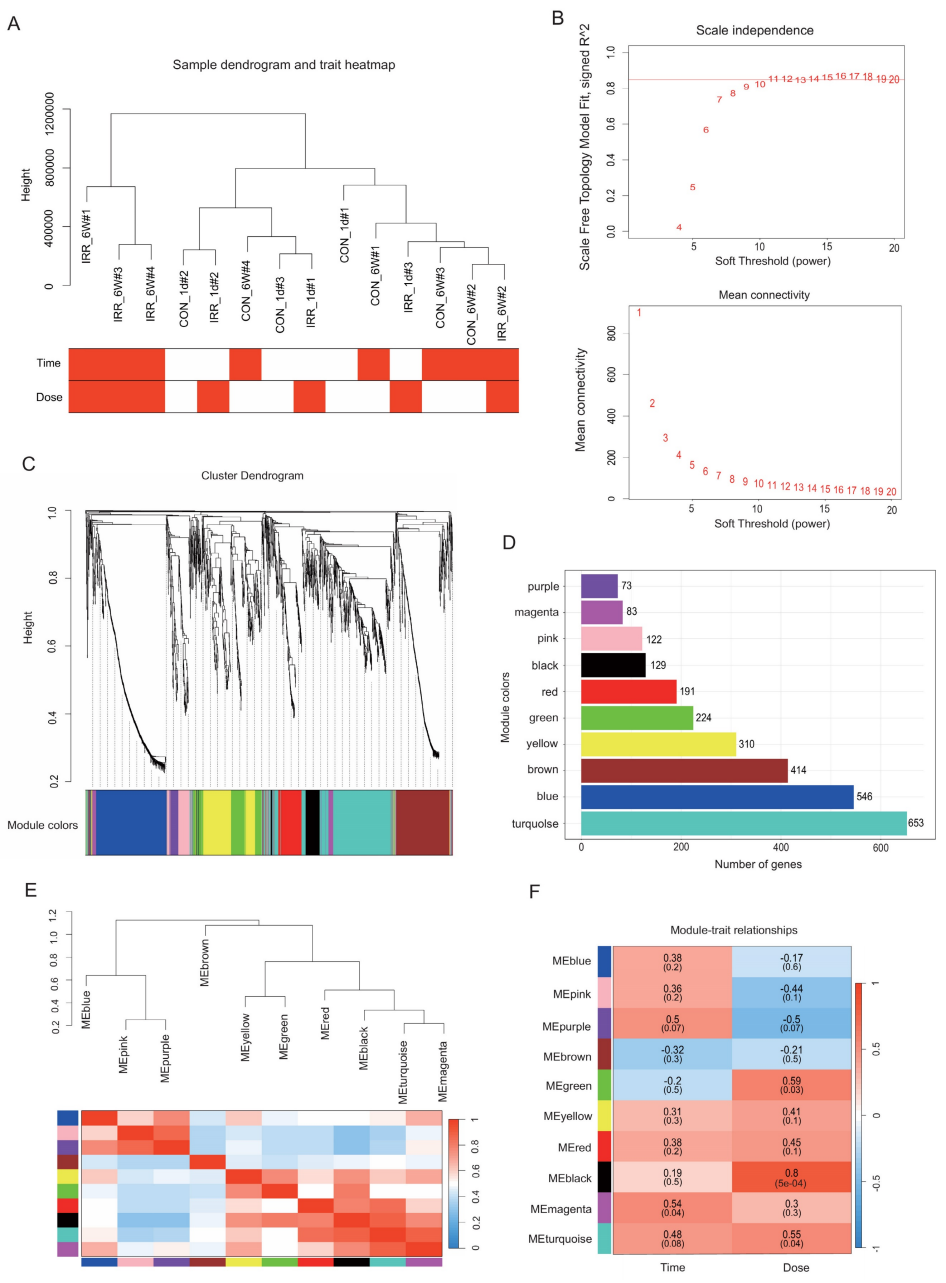
Data preparation of co-expression network. **A** Radiation condition heatmap and hierarchical clustering dendrogram. The degree of heatmap from white to dark red recognizes low to high levels of radiation condition. The radiation condition includes time and dose. **B** A scale-free co-expression network was estimated using soft-thresholding powers, with the best power value β determined to be 11. **C** Eleven co-expression modules constructed by clustering dendrograms and partitioned into different module colors (non-clustering genes shown in grey). **D** The bar plot of numbers in modules. **E** Hierarchical clustering trees and heatmap of different modules. The red shows positive correlation and blue shows negative correlation. **F** Correlation relationship between each network module and conditions. The values in the matrix cell indicate the correlation coefficient and the related *P*-value.

Using the WGCNA R package, a gene co-expression network analysis was conducted on the 2,774 DEGs. Gene expression values from all samples were analyzed through sample clustering and phenotypic heat map techniques. Fig. 4A indicates the absence of notable outliers, allowing the inclusion of all samples in the following data analysis. Based on the scale-free network fit index and average connectivity, the dataset's soft threshold was determined and chosen to be β = 8 (Fig. 4B). Next, the proximity and TOM matrices among genes were computed, followed by the creation of a hierarchical clustering dendrogram of the genes using the TOM matrix (Fig. 4C) 17. Later, the genes were categorized into ten groups through the dynamic shearing tree technique. The figure displays 10 gene modules represented by colored rectangles with the gene occupancy ratio on the vertical axis. Genes are grouped into color-coded modules, with each leaf on the tree representing a gene (Fig. 4C and D). The following are the module names along with their respective gene counts: purple (73), magenta (83), pink (122), black (129), red (191), green (224), yellow (310), brown (414), blue (546), and turquoise (653). Supplementary Table S1 contains the genes from each module.

### Correlation between modules with irradiation time and dose

The heatmap in Fig. 4E displays the correlations between gene modules and irradiation dose and time. In order to generate the map, the association between each gene module and irradiation characteristics was computed, followed by hierarchical clustering and heatmap analyses for every module 17. In this study, we focused on the black module, which exhibited the highest positive correlation (r = 0.8, *P* = 5e-4) with the irradiation trait, and the magenta module, which exhibited the highest positive correlation with the time phenotype (r = 0.54, *P* = 0.04) (Fig. 4F).

### Network visualization and functional enrichment study

**Figure 5:**
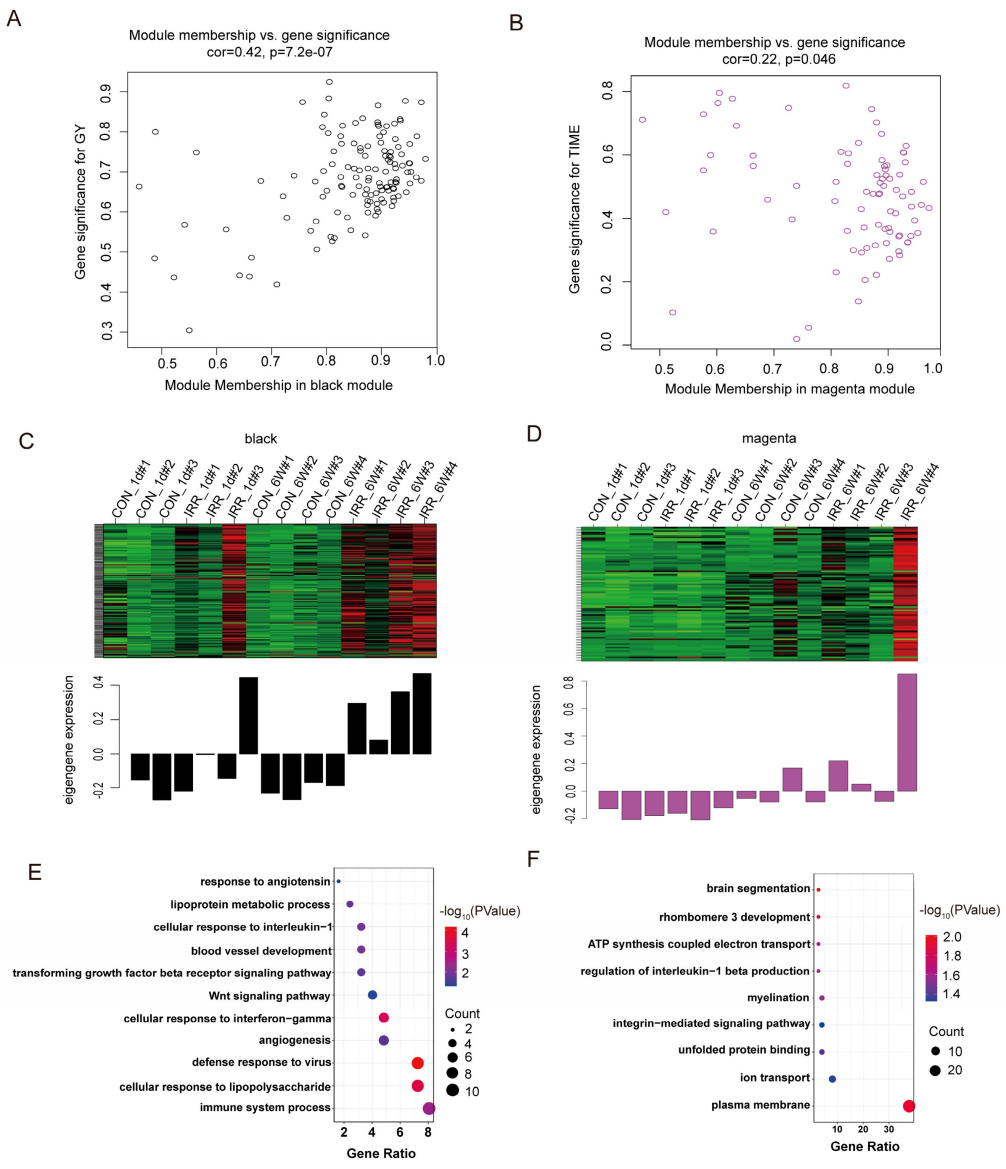
Correlation between modules with radiation traits. **A, B** Scatter plot in black **A** and magenta **B** modules with GS (y-axis) and MM (x-axis). **C, D** The expression level of genes in black **C** and magenta **D** modules. In the heatmap, green indicates the low expression and red indicates the high expression for samples. **E** GO analysis of biological process of black module. **F** GO analysis of biological process of magenta module.

The black and magenta modules were selected as key modules for intramodular analysis, respectively. The correlation coefficient between GS and MM was r = 0.42, *P* < 0.0001 for the black module (Fig. 5A) and r = 0.22, *P* < 0.05 (Fig. 5B) for the magenta module. Fig. 5C and D illustrated the heatmap depicting eigengene expression across the two modules.

The size of the node indicates gene importance, while the thickness of the edge signifies the strength of the link between nodes 17. We conducted GO enrichment analysis on the genes within each of the two modules individually, and the genes corresponding to each module are displayed in Fig. 5E and 5F. As illustrated in Fig. 5E, the gene roles of the black module were associated with the immune system process (GO 0002376) and the cellular reaction to lipopolysaccharide (GO 0071222). Additionally, the gene roles within the magenta module were mainly concentrated in the plasma membrane (GO 0005886) and the control of ion transport (GO 0006811) (Fig. 5F).

**Figure 6:**
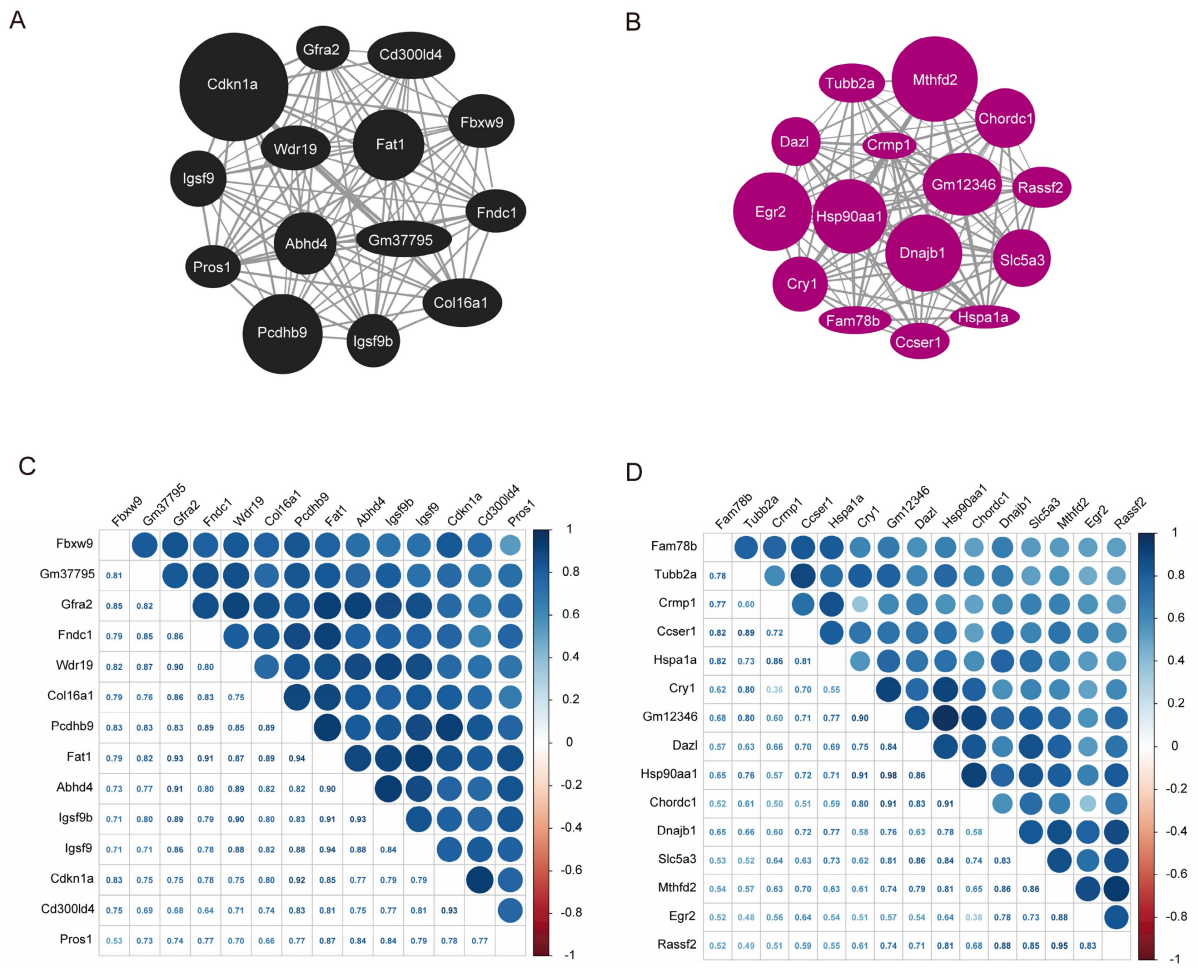
Hub gene map and heatmap of co-expression correlation between the hub genes. **A** Hub gene map of the black module. **B** Hub gene map of the magenta module. **C** Heatmap of co-expression correlation between hub genes in black module. **D** Heatmap of co-expression correlation between hub genes in magenta module. The darker blue in the upper right part indicates the stronger correlation, and the data in the lower left part are the correlation coefficients.

A graph depicting the interaction between the black and magenta modules within the co-expression network genes were created based on the gene weights. Within the black module, *Cdkn1a, Pcdhb9, and Fat1* are recognized as central genes (Fig. 6A). In contrast, *Mthfd2, Dnajb1, Hsp90aa1, Egr2* and *Gm12346* were identified as key genes in the magenta module (Fig. 6B). The co-expression correlation heatmap between hub genes in the black module and magenta module was drawn, and the darker blue in the upper right part indicated a stronger correlation, while the data in the lower left part represent the correlation coefficients. As demonstrated in Fig. 6C and D, the correlation coefficients among the majority of genes in these two modules were > 0.65, indicating a high correlation between the hub genes in the modules and a close linkage between their upstream and downstream regulatory roles.

### Validation of related genes by immunohistochemistry

**Figure 7:**
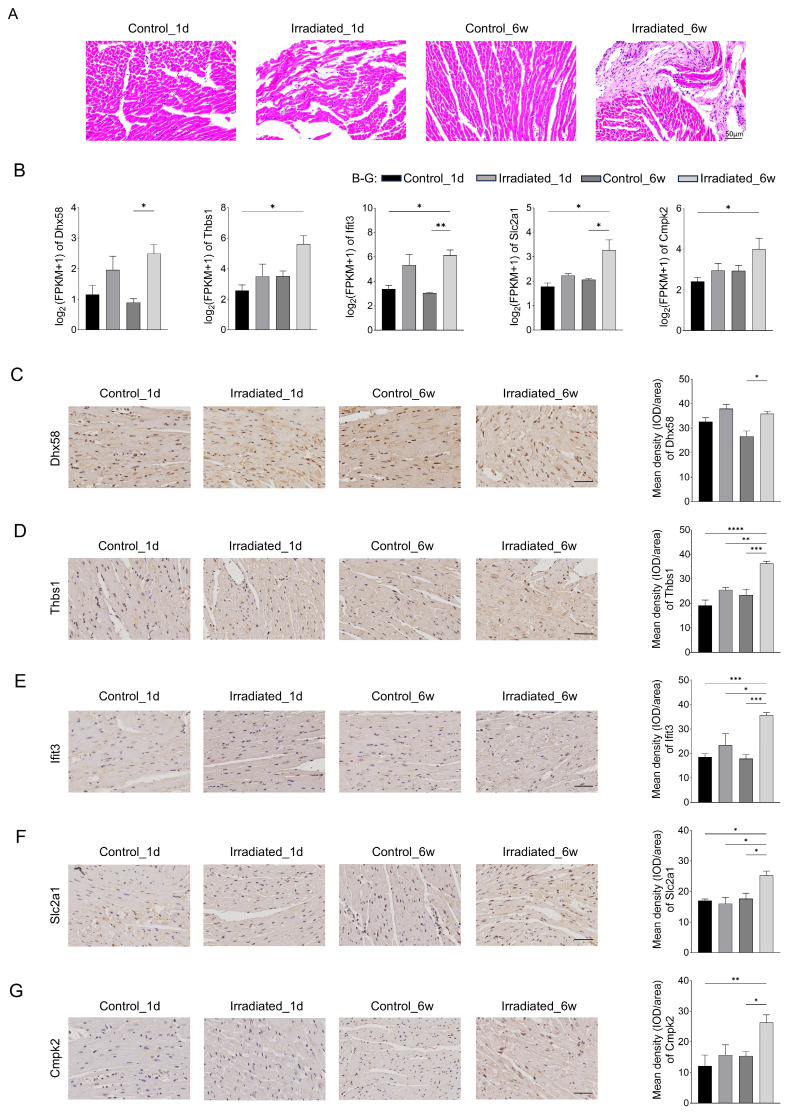
Validation of related genes by immunohistochemistry. **A** Representative plots of 4 groups of myocardial tissue after H&E staining. Scale bar, 50 μm. **B** Relative expression of *Dhx58, Thbs1, Ifit3, Slc2a1*, and *Cmpk2* gene by transcriptome sequencing. **C** Relative expression of *Dhx58* in myocardial tissues of each group and their representative pictures. **D** Relative expression of *Thbs1* in myocardial tissues of each group and their representative pictures. **E** Relative expression of *Ifit3* in myocardial tissues of each group and their representative pictures. **F** Relative expression of *Slc2a1* in myocardial tissues of each group and their representative pictures. **G** Relative expression of *Cmpk2* in myocardial tissues of each group and their representative pictures. Scale bar, 50 μm.

Morphological analysis was also conducted through hematoxylin-eosin staining to evaluate radiation-induced injury in heart tissues. Fig. 7A showed that myocardial damage can be induced one day after irradiation, accompanied by inflammatory cell infiltration. The damage was more severe in six weeks after irradiation, with increased inflammatory cells and fibrosis. Six weeks post-irradiation, RNA-seq analysis revealed that the experimental group exhibited markedly elevated levels of *Dhx58, Ifit3*, and *Slc2a1* in comparison to the control group (Fig. 7B). Moreover, the experimental group exhibited elevated levels of *Thbs1* and *Cmpk2* expression, though the increase was not statistically meaningful. Fig. 7C, F and G showed the results of our immunohistochemical analysis of cardiac tissue. *Dhx58*, *Slc2a1*, and *Cmpk2* expression were significantly higher in the experimental group six weeks after irradiation (*P* <0.05). Additionally, the experimental group exhibited a markedly higher expression of *Thbs1* and *Ifit3* compared to the control group (*P* < 0.0001) (Fig. 7D and E).

## Discussion

This study found that acute radiation myocardial injury is associated with inflammation, while chronic radiation myocardial injury is associated with myocardial fibrosis. The study also identified *Cmpk2, Ifit3, Dhx58, Slc2a1*, and *Thbs1* as being associated with RIHD. The potential mechanisms are systemic inflammation and mitochondrial dysfunction. These discoveries offer both theoretical and experimental foundations for understanding the mechanism and early detection of RIHD.

RIHD is classified into acute and chronic injury. Acute cardiac injury is characterized by endothelial damage resulting in vasodilation and increased vascular permeability within minutes to hours of ionizing radiation exposure. Injured endothelial cells release adhesion molecules and growth factors, which stimulate the production of tumor necrosis factor (TNF) and interleukins (IL-1 and IL-6), thereby enhancing the acute inflammatory response 18. Acute-phase inflammatory cells are predominantly neutrophils and are distributed in the myocardium in the area of radiotherapy 19. Our research noted that inflammatory cells infiltrated the heart tissues of mice within a day post-irradiation, unlike the control group, aligning with existing literature. Chronic changes in radiological cardiac injury, except for inflammation, are also closely related to chronic oxidative stress, mitochondrial dysfunction, and necrosis of cardiomyocytes, which are mainly characterized by increased myocardial fibrosis 20-22. The study found no myocardial fibrosis in the myocardial tissues of mice one day after irradiation. However, more pronounced myocardial fibrosis was observed six weeks after irradiation, which is consistent with the literature.

*Cmpk2* is primarily located in the mitochondria, and its loss of function causes mitochondrial deficiency 23. *Cmpk2* is actively triggered by various viral infections, including hepatitis E virus, HIV, and dengue virus 24, 25. Zhong *et al.* performed research uncovering the essential functions of *Cmpk2* in LPS-triggered inflammation by activating the inflammasome pathway 26. However, the role of *Cmpk2* in RIHD has not yet been investigated.

*Ifit3* is a gene responsible for encoding a protein, produced as a result of an interferon-stimulated gene. It plays a role in natural immunity, defense against viruses, and inflammation. Recent research indicates that IFIT contributes to the development of various cancers, such as oral cancer 27. Lu and colleagues showed that *Ifit1* and *Ifit3* play a role in LOXL2-driven migration, invasion, EMT, and CSC-like characteristics in oral squamous cell carcinomas 28. Chen *et al.* discovered that the *Ifit3* gene could potentially act as a biomarker for the diagnosis and treatment of ischemic cardiomyopathy 29. The research additionally assessed the impact on the mitochondria-associated factor voltage-dependent anion channel 2 (VDAC2) and apoptosis 30. Furthermore, although the *Ifit3* gene has been identified as one of the key genes related to heart failure, its role in RIHD has not yet been investigated 31.

*Dhx58*, which is alternatively called *Lgp2*, belongs to the family of retinoic-acid-inducible gene (RIG)-like receptors (RLRs). These receptors, known as pattern recognition receptors (PRRs), initiate a natural immune reaction to combat viral infections 32, 33. *Dhx58* is crucial for detecting viral infections 34. Zhao *et al.* found that DEAD-box helicase 5 (DDX5) reduced the mRNA expression of interferon beta (IFN-β), IL-6, and Dhx58 through the METTL3-METTL14/YTHDF2 pathway. The research showed that DDX5 interacted with antiviral transcripts and controlled immune reactions via YTHDF2-mediated mRNA degradation 35. Earlier research indicates that the gene responsible for this protein can activate macrophages to send signals prompting mitochondria to form inflammasomes. These inflammasomes generate proteins involved in the inflammatory response, contributing to the body's defense mechanism 36. However, the role of this protein in RIHD remains unclear.

The *Slc2a1* gene produces a glucose transporter responsible for controlling glucose absorption. It can increase intracellular glucose levels, providing a favorable environment for tumor growth, dissemination, and metastasis. Consequently, it is essential for the expansion and multiplication of cancer cells 37. Earlier research has indicated that elevated levels of *Slc2a1* correlate with poorer outcomes in lung, breast, and stomach cancers 38-40. Mouton *et al.* demonstrated an association between *Slc2a1* and polarization phenomena in resident macrophages and monocyte-derived macrophages following myocardial infarction 41. However, the role of *Slc2a1* in RIHD has not yet been investigated.

The *Thbs1* gene produces a component of a disulfide-bonded homotrimeric protein, an adhesive glycoprotein facilitating interactions between cells and between cells and the extracellular matrix 42. *Thbs1* influences inflammation through NF-κB pathways and is capable of initiating TGF-β1 signaling 43, 44. In myocardial injury, silencing *Thbs1* inhibits inflammatory cytokines, reduces oxidative stress and thus lowers cell apoptosis in vitro 45. Earlier research has indicated that *Thbs1* plays a role in the development of several heart conditions, such as septic cardiomyopathy, cardiac hypertrophy, fatal cardiac atrophy, and damage to heart cells caused by coxsackievirus B3 42, 46-48. However, its role in RIHD has not been reported.

The mechanisms of RIHD remain unclear, most studies suggest that it is the result of a combination of mechanisms, such as systemic inflammation and mitochondrial dysfunction 49, 50. Firstly, radiation increases oxidative stress, directly causing damage to macromolecules such as DNA, proteins and lipids 14, 51. In addition, radiation can directly damage the respiratory chain of mitochondria, leading to mitochondrial dysfunction, which leading to ATP production decreased, ROS production increased, and ultimately apoptosis 12. Excessive ROS cause endothelial cell damage, prompting them to secrete adhesion molecules and growth factors, triggering an acute inflammatory response 21. Myocardial fibrosis is considered to be the final stage of RIHD and is characterized by excessive collagen deposition in damaged cardiac tissues as a result of multiple pathways involving inflammation, oxidative stress, and chronic changes in gene expression 52. Our research revealed that the core genes were associated with the immune system process (GO 0002376), cellular reaction to lipopolysaccharide (GO 0071222), plasma membrane (GO 0005886), and ion transport regulation (GO 0006811). Furthermore, the genes such as *Cmpk2, Ifit3, Dhx58*, and *Thbs1* are linked to both systemic inflammation and mitochondrial dysfunction, as supported by existing literature.

This study has several potential limitations. Firstly, the sample size is small. Secondly, in this study, only immunohistochemistry was used for preliminary validation, and the mechanism was not explored in depth. Additional studies both in the lab and in living organisms are required for future confirmation. Thirdly, the mice involved in this study were all male and could not be analyzed for sex differences.

## Conclusions

Acute radiation myocardial injury is characterized by inflammation, while chronic radiation myocardial injury is characterized by myocardial fibrosis. RIHD is associated with* Cmpk2, Ifit3, Dhx58, Slc2a1*, and *Thbs1* genes, which may be potential targets for early diagnosis and intervention in RIHD.

## Supplementary Material

Supplementary table 1.

Supplementary table 2.

Supplementary table 3.

## Abbreviations

RIHD: radiation-induced heart disease
CAD: coronary artery disease
DEGs: differentially expressed genes
WGCNA: weighted gene co‑expression network analysis
PCA: principal component analysis
MEs: module eigengenes
GS: gene significance
MM: module membership
GO: gene ontology
KEGG: kyoto encyclopedia of genes and genomes
H&E: hematoxylin and eosin
DAB: diaminobezidin 3
TNF: tumor necrosis factor
IL-1: interleukin-1
IL-6: interleukin-6
DENV: dengue virus
LPS: lipopolysaccharide
LOXL2: lysyl oxidase-like protein 2
OSCC: oral squamous cell carcinomas
EMT: epithelial-mesenchymal transition
CSC: cancer stem cell
VDAC2: voltage dependent anion channel 2
RIG: retinoic- acid-inducible gene
RLRs: retinoic- acid-inducible gene (RIG)-like receptors
PRRs: pattern recognition receptors
DDX5: DEAD-box helicase 5
IFN-β: interferon beta
TGF-β1: transforming growth factor-beta
